# Effects of 1,25-Dihydroxyvitamin D_3_ and 25-Hydroxyvitamin D_3_ on PBMCs From Dairy Cattle Naturally Infected With *Mycobacterium avium* subsp. *paratuberculosis*

**DOI:** 10.3389/fvets.2022.830144

**Published:** 2022-02-08

**Authors:** Taylor L. T. Wherry, Shankumar Mooyottu, Judith R. Stabel

**Affiliations:** ^1^Infectious Bacterial Diseases, National Animal Disease Center, United States Department of Agriculture-Agricultural Research Service, Ames, IA, United States; ^2^Department of Veterinary Pathology, Iowa State University, Ames, IA, United States

**Keywords:** *Mycobacterium avium* subsp. *paratuberculosis*, cattle, vitamin D, PBMC, immune responses, Johne's disease

## Abstract

The role of vitamin D_3_ in modulating immune responses has been well-established for over two decades; however, its specific functions have not been extensively detailed in cattle, particularly cattle in different stages of infection with *Mycobacterium avium* subspecies *paratuberculosis* (MAP). Consistent with previous work in our lab, the present study showed that infected cattle in the clinical stage of disease have reduced serum 25-hydroxyvitamin D_3_ [25(OH)D_3_]. Additionally, effects of vitamin D_3_ on peripheral blood mononuclear cells (PBMCs) from naturally infected dairy cattle in subclinical (*n* = 8) or clinical (*n* = 8) stages of infection were compared to non-infected control cows (*n* = 8). Briefly, PBMCs were isolated and cultured *in vitro* with 4 ng/ml 1,25-dihydroxyvitamin D_3_ [1,25(OH)_2_D_3_] or 100 ng/ml 25(OH)D_3_. Treatment with 1,25(OH)_2_D_3_ resulted in decreased secretion for some pro-inflammatory cytokines in clinical animals, including IL-1β, IL-6, and IFN-γ. Similar responses for IL-1β and IL-6 were noted with the addition of 25(OH)D_3_. Additionally, pro-inflammatory cytokine gene expression tended to be upregulated in PBMCs from clinical animals after treatment with 1,25(OH)_2_D_3_. In contrast, PBMCs from clinical animals treated with 25(OH)D_3_ showed downregulation of pro-inflammatory cytokine gene expression, although only significant for *IL1B*. Following 25(OH)D_3_ treatment, clinical animals showed significant reduction in CD4+CD25+ T cells. *CYP27B1* gene expression was notably decreased in clinical and control animals following 25(OH)D_3_ treatment but increased in subclinical cows. 1,25(OH)_2_D_3_ treatment reduced *CYP24A1* gene expression in all groups, while 25(OH)D_3_ treatment only significantly reduced expression for control cows. Lastly, serum 25(OH)D_3_ levels were significantly lower in clinical animals. Taken together, these data show vitamin D_3_ modulates cytokine signaling in cattle at different stages of MAP infection and, therefore, may have implications on disease progression.

## Introduction

Vitamin D_3_ has classically been acknowledged for its role in calcium regulation and bone homeostasis (1); however, more recent studies have revealed its role in regulating innate and adaptive immune responses to infectious pathogens (2). Vitamin D_3_ is found within the body in two forms. The inactive analog, 25-hydroxyvitamin D_3_ [25(OH)D_3_], can be commonly found in the circulation bound to its vitamin-D-binding protein. This form can be taken up by host immune cells, including T cells (3) and antigen presenting cells (APCs) (4–6), then converted via hydroxylation by 1α-hydroxylase (CYP27B1) to its biologically active analog, 1,25(OH)_2_D_3_ (1).

Limited work has been done to provide a solid foundation for the role that vitamin D_3_ may play in modulating the immune response to infectious pathogens, particularly in cattle. Previous reports have demonstrated the ability of 1,25(OH)_2_D_3_ to upregulate gene expression of inducible nitric oxide synthase (iNOS/*NOS2*), IL-1β (*IL1B*), 24-hydroxylase (*CYP24A1*), and Regulated Upon Activation, Normal T cell Expressed and Secreted (RANTES/*CCL5*) in bovine monocytes isolated from healthy dairy cows (4). A decrease in antigen-specific IFN-γ responses has also been observed in peripheral blood mononuclear cells (PBMCs) from *Mycobacterium bovis* (*M. bovis*) infected cattle following treatment with 1,25(OH)_2_D_3_ (7). Moreover, a study on *Streptococcus uberis* induced acute mastitis showed 25(OH)D_3_ reduced both the mammary gland bacterial load and clinical symptoms following treatment of the infected mammary tissue with 100 μg 25(OH)D_3_ after each milking (8).

Modulation of immune responses by vitamin D_3_ has been established in human models. Peripheral blood mononuclear cells from *Mycobacterium tuberculosis* (*M. tb*) patients treated with 1,25(OH)_2_D_3_ exhibit a significant reduction in Th1 pro-inflammatory cytokines IFN-γ, IL-12p40, and IL-6 with a concomitant decrease in expression of IL-10 (9). An additional study of *M. tb* infection in humans demonstrated enhanced antimicrobial activity of alveolar macrophages upon treatment with 1,25(OH)_2_D_3_ (6).

Previous work in our lab has shown cattle at the clinical stage of *Mycobacterium avium* subspecies *paratuberculosis* (MAP) infection possess significantly decreased levels of circulating 25(OH)D_3_ compared to uninfected and subclinical cows (10). Additionally, animals humanely euthanized in this study due to severe clinical disease symptoms had a 20% reduction in their serum 25(OH)D_3_ concentrations. Furthermore, insufficient levels of circulating 25(OH)D_3_ have been associated with an increased risk of disease severity (11–13) and susceptibility to autoimmune disorders (14–16).

Little work has been done thus far on the outcomes of exogenous vitamin D_3_ treatment on immune function and cell receptor expression in cattle infected with MAP. In the present study, we hypothesized that addition of 1,25(OH)_2_D_3_ or 25(OH)D_3_ to PBMCs isolated from dairy cattle naturally infected with MAP would modulate antigen specific inflammatory responses to a whole cell sonicate of MAP. To test this, our objectives were to measure differences in inflammatory cytokine secretion and gene expression responses, along with differences in cell surface marker expression.

## Materials and Methods

### Animals

Animals used in this study were Holstein dairy cows ranging in age from 2 to 11 years old. Cows were stratified into infection status groups based on results from diagnostic tests measuring serum MAP-specific antibody levels (Herdchek; IDEXX, Westbrook, ME), bovine IFN-γ plasma levels (Bovigam; Prionics, La Vista, NE), and fecal shedding detected by culture on Herrold's egg yolk medium (Becton Dickinson, Sparks, MD) as previously described (17). Clinical cows (*n* = 8) were characterized as being ELISA positive for MAP serum antibody, with an average S/P ratio of 1.36, and had a MAP-specific IFN-γ recall response of OD_450_ 0.43 ± 0.22 (Abs_450nm_MPS-Abs_450nm_NS). This was the only group that was culture positive for MAP fecal shedding. Subclinical cows (*n* = 8) were ELISA negative for MAP serum antibodies and IFN-γ OD_450_ results averaged 0.15 ± 0.05. Animals assigned to the control group (*n* = 8) were negative for all MAP diagnostic tests.

All animals were housed in American Association for Accreditation of Laboratory Animal Care-accredited facilities, and all animal related procedures were approved by the IACUC (National Animal Disease Center, Ames, IA). To prevent cross-contamination between groups, infected cows, and healthy control cows were housed separately on-site. A total mixed ration (TMR) was fed to all cows, which was comprised of corn silage, chopped hay, cracked corn, and soybean meal. Additionally, dietary supplementation with vitamin D_3_ yielded an estimated intake of 40,000 IU per day per animal. Cows were either dry or at various stages of lactation at the time of sample collection.

### Vitamin D_3_ Stock Preparation

Stocks of 25(OH)D_3_ and 1,25(OH)_2_D_3_ were prepared in pure ethanol and stored in airtight glass vials at −20°C and kept protected from light at all times. Final ethanol concentrations for 25(OH)D_3_ and 1,25(OH)_2_D_3_ treatments did not exceed 0.11 and 0.05%, respectively.

### Serum 25(OH)D_3_ Quantitation

Whole blood was collected using serum separation vacutainer tubes (Becton Dickinson, Franklin Lakes, NJ), allowed to clot, and centrifuged 872 × g (2,000 RPM) for 30 min at room temperature. Serum samples were transferred to 1.5 ml microcentrifuge tubes (Axygen, Union City, CA) and stored at −80°C. Concentrations of 25(OH)D_3_ were measured by liquid chromatography with tandem mass spectrometry (LC/MS/MS; Heartland Assays, Ames, IA).

### PBMC Isolation and Culture

Whole blood was harvested via jugular venipuncture into 2 × acid-citrate-dextrose (in-house, 1:10). Peripheral blood mononuclear cells were isolated from the buffy coat fraction using a Histopaque-1077 (Sigma) density gradient. Peripheral blood mononuclear cells were then resuspended in complete medium [cRPMI; RPMI-1640 with L-glutamine and HEPES (Gibco, Grand Island, NY), 1% antibiotic-antimycotic (100 U/ml penicillin, 100 μg/ml streptomycin, 250 ng/ml Amphotericin B, Gibco), 1% MEM non-essential amino acids solution (100 × , Gibco), 2% MEM essential amino acids solution (50 × , Gibco), 2 mM L-glutamine (200 mM, Gibco); 1% sodium pyruvate (100 mM, Gibco); and 50 μM 2-mercaptoethanol (50 mM, Gibco)] supplemented with 10% (v/v) heat inactivated fetal bovine serum (FBS, HyClone Cytiva, Marlborough, MA). Cell viability and quantity were determined using trypan blue exclusion on a TC20 automated cell counter (Bio-Rad, Hercules, CA) and cell concentrations were adjusted to 4.0 × 10^6^ viable cells per ml in cRPMI with 10% FBS.

Peripheral blood mononuclear cells were added to 24-well flat-bottom plates (Becton Dickinson) at 0.5 ml per well with 1.0 ml control media (NS; non-stimulated) or treatment media containing 1.0 μg/ml lipopolysaccharide (LPS; Sigma-Aldrich) or 10 μg/ml whole-cell sonicate of (MPS) in cRPMI ± 25(OH)D_3_ or 1,25(OH)_2_D_3_. Peripheral blood mononuclear cells were harvested from cows at two time points and treatment with either form of vitamin D_3_ represents two separate experiments. Vitamin D_3_ treatment concentrations were selected based on previous work in cattle (4, 5). Plates were incubated for 24 h in a 39°C humidified incubator then centrifuged 500 × g for 10 min. Supernatants were collected and stored in 1.5 ml microcentrifuge tubes (Axygen) at −80°C until analyses were performed. The remaining cells in each well were dislodged with cold PBS and a 15 min incubation on ice, then transferred to separate 1.5 ml microcentrifuge tubes (Axygen) and stored in 350 μl RNAprotect Cell Reagent (Qiagen, Hilden, Germany) at −80°C until RNA extraction.

Peripheral blood mononuclear cells were also added to two sets of 48 well flat-bottom plates (Corning Inc.) at 0.2 ml per well with 0.5 ml media with a final concentration of 10% FBS and contained NS control media or 10 μg/ml MPS incubated for 6 days. Duplicates of each treatment were set up for addition of ± 25(OH)D_3_ or 1,25(OH)_2_D_3_ and all plates were incubated at 39°C in a humidified incubator.

### Flow Cytometry

On day 6 cells were centrifuged at 500 × g for 5 min to remove supernatant, resuspended in D-PBS, pH 7.4, then transferred to a 96 well plate in preparation for staining with Zombie Yellow fixable viability dye (BioLegend, San Diego, CA) and surface labeling with antibodies. Following labeling with primary, secondary, and directly conjugated antibodies in Table 1, cells were resuspended in 200 μl stabilizing fixative and analyzed using a BD LSR II flow cytometer (BD Biosciences, San Jose, CA). Data were analyzed using FlowJo software (Tree Star, Inc., San Carlos, CA) and cell populations were expressed as a percentage of the live cell population.

**Table 1:** Flow cytometric analysis antibody panels.

**Panel**	**Target**	**Clone**	**Source**	**Fluorochrome**	**Secondary isotype**
1	CD4	ILA11A	Washington State, Pullman, WA	BB700	IgG2a
1	CD25	LCTB2A	Washington State, Pullman, WA	BUV395	IgG3
1	CD26	CC69	Serotec, Oxford, UK	APC (direct)	n/a
1	CD40	ILA156	USBiological, Salem, MA	MaxLight 405 (direct)	n/a
1	CD45RO	ILA116	Serotec, Oxford, UK	PE (direct)	n/a
1	IgM (B cell)	PIG45A2	Washington State, Pullman, WA	AF350	IgG2b
1	CD86	ILA190	Serotec, Oxford, UK	PE Cy7	IgG1
2	CD8	7C2B	Washington State, Pullman, WA	BB700	IgG2a
2	CD25	LCTB2A	Washington State, Pullman, WA	BUV395	IgG3
2	CD26	CC69	Serotec, Oxford, UK	APC (direct)	n/a
2	CD40	ILA156	USBiological, Salem, MA	MaxLight 405 (direct)	n/a
2	CD45RO	ILA116	Serotec, Oxford, UK	PE (direct)	n/a
2	gdTCR	GB21A	Washington State, Pullman, WA	AF350	IgG2b
2	CD86	ILA190	Serotec, Oxford, UK	PE Cy7	IgG1

### PBMC RNA Extraction and cDNA Synthesis

Peripheral blood mononuclear cell samples stored in RNAprotect Cell Reagent (Qiagen), described above, were thawed and centrifuged 5,000 × g for 5 min. The cell pellet was lysed using Buffer RLT Plus (Qiagen) supplied in the RNeasy Mini kit. On-column RNA purification was performed according to the kit instructions and eluted in a final volume of 40 μl RNase-free water also supplied in the kit. Total RNA was quantified using the RNA 6000 Nano kit (Agilent, Santa Clara, CA) and the 2100 Bioanalyzer instrument (Agilent). Approximately 86% of samples for the 25(OH)D_3_ experiment had RIN > 9.0, and the lowest RIN was 8.1. Additionally, about 85% of samples for the 1,25(OH)_2_D_3_ experiment had RIN > 9.0 with the lowest RIN measured at 7.9. Purified RNA was diluted to 12.5 ng/μl in 40 μl RNAse-free water. Superscript IV (Invitrogen, Carlsbad, CA) was used to reverse transcribe RNA. The reaction mixture included a final concentration of 175 ng random hexamer primers (Invitrogen), 600 nM of each dNTP (Invitrogen), and 2,000 units of Superscript IV. Primers were annealed for 5 min at 65°C, followed by incubation with the reverse transcriptase enzyme for 10 min at 23°C, 10 min at 50°C, then 10 min at 80°C per the manufacturer instructions. For storage at −20°C, cDNA was diluted 1:10 in RNase and DNase free water (Gibco).

### Cytokine Gene Expression Real-Time qPCR

TaqMan bovine gene expression assays (Applied Biosystems, Foster City, CA) listed in Table 2 were used to quantify relative expression of *IL1B* (IL-1β), *IL10* (IL-10), *IL12A* (IL-12A), *IL17A* (IL-17A), *DEFB7* (β-defensin 7), *DEFB10* (β-defensin 10), *CYP24A1* (24-hydroxylase), *CYP27B1* (1α-hydroxylase), *IFNG* (IFN-γ), *NOS2* (iNOS), *CCL5* (RANTES), and *TNF* (TNF-α) in 24 h PBMC culture samples. Samples were plated in duplicate with a reaction mixture consisting of 10 μl TaqMan Fast Advanced Master Mix (Applied Biosystems), 1 μl gene expression assay, 5 μl nuclease-free water, and 4 μl cDNA template per well. Relative quantitation (RQ) values were calculated by normalization to 18S rRNA expression (FAM/MGB probe, non-primer limited; Applied Biosystems) (17, 18) and calibration to the NS sample. Data were analyzed using the 2^−Δ*ΔCT*^ method (19).

**Table 2:** ThermoFisher scientific gene expression assays.

**Target**	**Gene alias**	**Assay ID**	**Target sequence**
IL-1β	*IL1B*	Bt03212742_m1	ACAGATGAAGAGCTGCATCCAACAC
IL-10	*IL10*	Bt03212725_g1	CTGGATGACTTTAAGGGTTACCTGG
IL-12A	*IL12A*	Bt03213918_m1	GCTACAGAAGGCCAGACAAACTCTA
IL-17A	*IL17A*	Bt03210251_m1	ACTTCATCTATGTCACTGCTACTGC
β-defensin 7	*DEFB7*	Bt04318496_mH	TGTCTGCTGGGTCAGGATTTACTCA
β-defensin 10	*DEFB10*	Bt03415224_m1	TGTCTTCTGGGTCAGGATTTACTCA
24-hydroxylase	*CYP24A1*	Bt04306549_g1	AAAGGAATTGTCCGCAAATACGACG
1α-hydroxylase	*CYP27B1*	Bt04311111_g1	GGATTGCTCACCGCGGAAGGGGAAG
IFN-γ	*IFNG*	Bt03212722_g1	ATTGGAAAGATGAAAGTGACAAAAA
iNOS	*NOS2*	Bt03249590_m1	CAGCCCCCGTCCAGTCCAGTGACAC
RANTES	*CCL5*	Bt03216832_m1	CTCCATGGCAGCAGTTGTCTTTATC
TNF-α	*TNF*	Bt03259155_g1	CAAACACTCAGGTCCTCTTCTCAAG

### Cytokine Secretion

Concentrations of secreted cytokines were quantified in cell culture supernatants using a custom Milliplex bovine 8-plex cytokine/chemokine magnetic bead panel consisting of IL-1β, IL-6, IL-10, IL-17A, IFN-γ, TNF-α, IL-36RA, and MCP-1. Samples were loaded onto 96-well plates in duplicate and incubated with beads overnight (16–18 h) at 4°C on a plate shaker protected from light. Samples were further processed the following day by the addition of biotinylated detection antibodies, followed by Streptavidin-Phycoerythrin. Unbound reagents were removed via manual washing utilizing a magnetic plate to retain beads. Beads were resuspended in Drive Fluid and cytokine concentrations were measured by running samples on the Luminex MAGPIX xMAP instrument. Bead data corresponding to specific cytokines was aggregated based on pre-determined internal bead dye ratios, therefore individual cytokines were identified via specific fluorescent signal. Cytokine concentrations were measured by Phycoerythrin fluorescence compared to a standard curve. Data were collected and summarized using the Bio-Plex Manager software (Bio-Rad).

IL-12 concentrations were quantified by standard ELISA using a capture antibody to the p40 subunit (MCA1782EL, Bio-Rad) and biotinylated detection antibody (MCA2173B, Bio-Rad) at concentrations of 1.1 and 0.5 μg/ml, respectively. A standard curve was generated using a bovine IL-12/IL-23 p40 recombinant protein (RP0077B; Kingfisher, Saint Paul, MN). Colorimetric changes were developed through the addition of HRP bound streptavidin (AR0068-001, Kingfisher), followed by incubation with TMB substrate. The reaction was terminated using stop solution (AR0133-002; Kingfisher). Plates were read within 5 min and signal was detected at 450 nm using a SpectraMax 340PC384 microplate reader (Molecular Devices, San Jose, CA).

### Statistical Analysis

Statistical analysis was performed using R Statistical Software (version 4.0.3, R Foundation for Statistical Computing, Vienna, Austria) and RStudio (version 1.3.1093, Boston, MA). Statistical models were built for cytokine experiments using the mixed model function “lme” from package “nlme” (20) and flow cytometry experiments using the mixed model function “lmer” from package “lme4” (21). Serum 25(OH)D_3_ concentration data were analyzed by ANOVA. All *post-hoc* tests were performed using the package “emmeans” (22) with a Tukey adjustment for multiple comparisons. ΔΔCt values were used for relative gene expression analysis and data were transformed for presentation and averaged using the 2^−Δ*ΔCt*^ method (19).

## Results

### Serum 25(OH)D_3_ Concentrations

**Figure 1:**
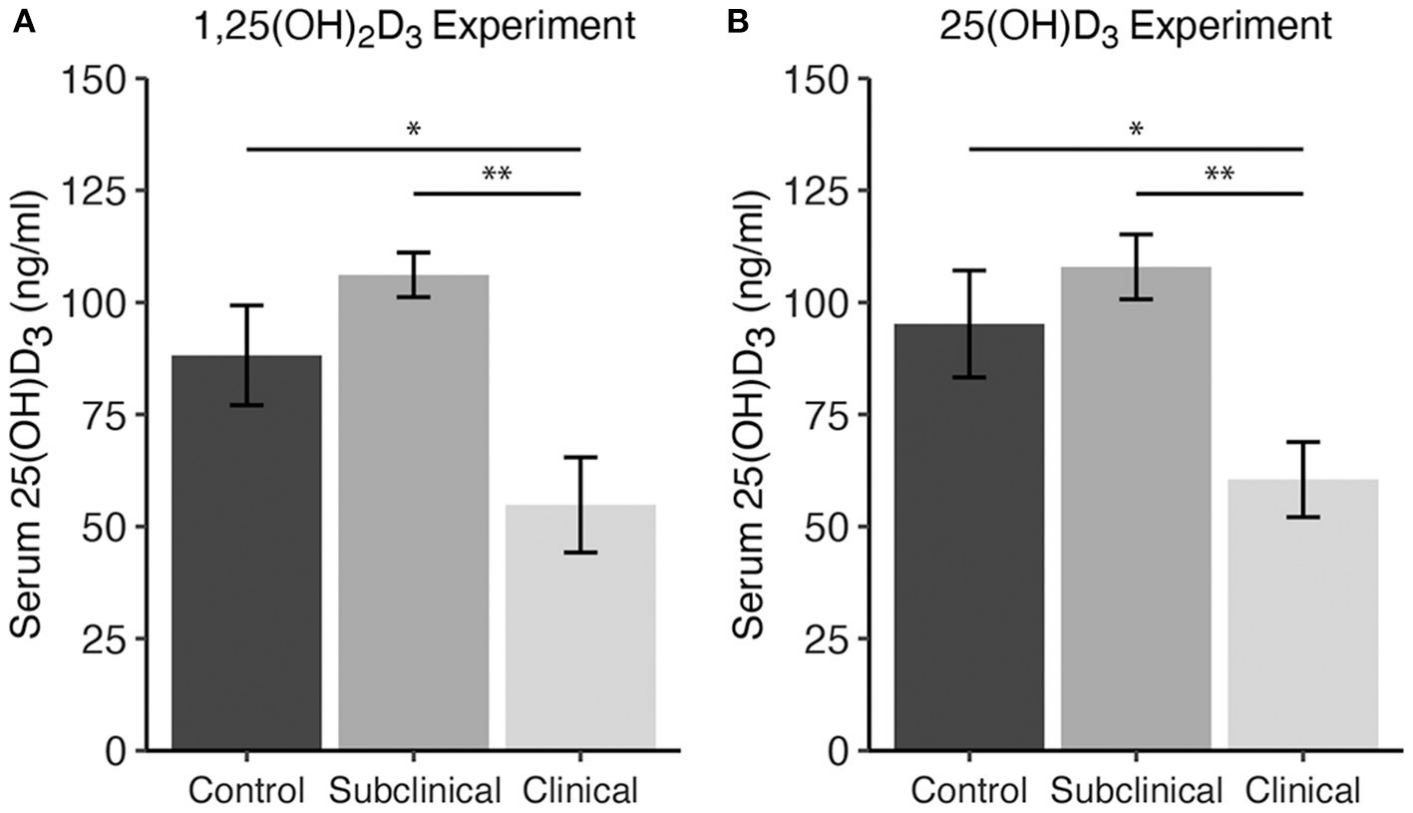
Serum 25-hydroxyvitamin D_3_ concentrations (ng/ml) for dairy cattle grouped by different stages of infection with *Mycobacterium avium* ssp. *paratuberculosis* for two separate experiments including either **(A)** 1,25(OH)_2_D_3_ or **(B)** 25(OH)D_3_ treatment. Each infection status group is comprised of *n* = 8. Whole blood was collected via jugular venipuncture into serum separation vacutainer tubes, allowed to clot, and centrifuged to collect serum. Data are presented as the mean ± SE and significance levels are as follows: * < 0.05, ** < 0.01.

Total 25(OH)D_3_ concentration in serum samples from each cow were analyzed for each vitamin D_3_ treatment experiment. Averages across cows in each infection status group revealed reduced 25(OH)D_3_ levels in clinically infected animals (Figure 1). This was significant for both experiments upon comparison with subclinical cows (*P* < 0.01) and non-infected controls (*P* < 0.05).

### Cytokine Secretion Following 1,25(OH)_2_D_3_ or 25(OH)D_3_ Treatment

**Figure 2:**
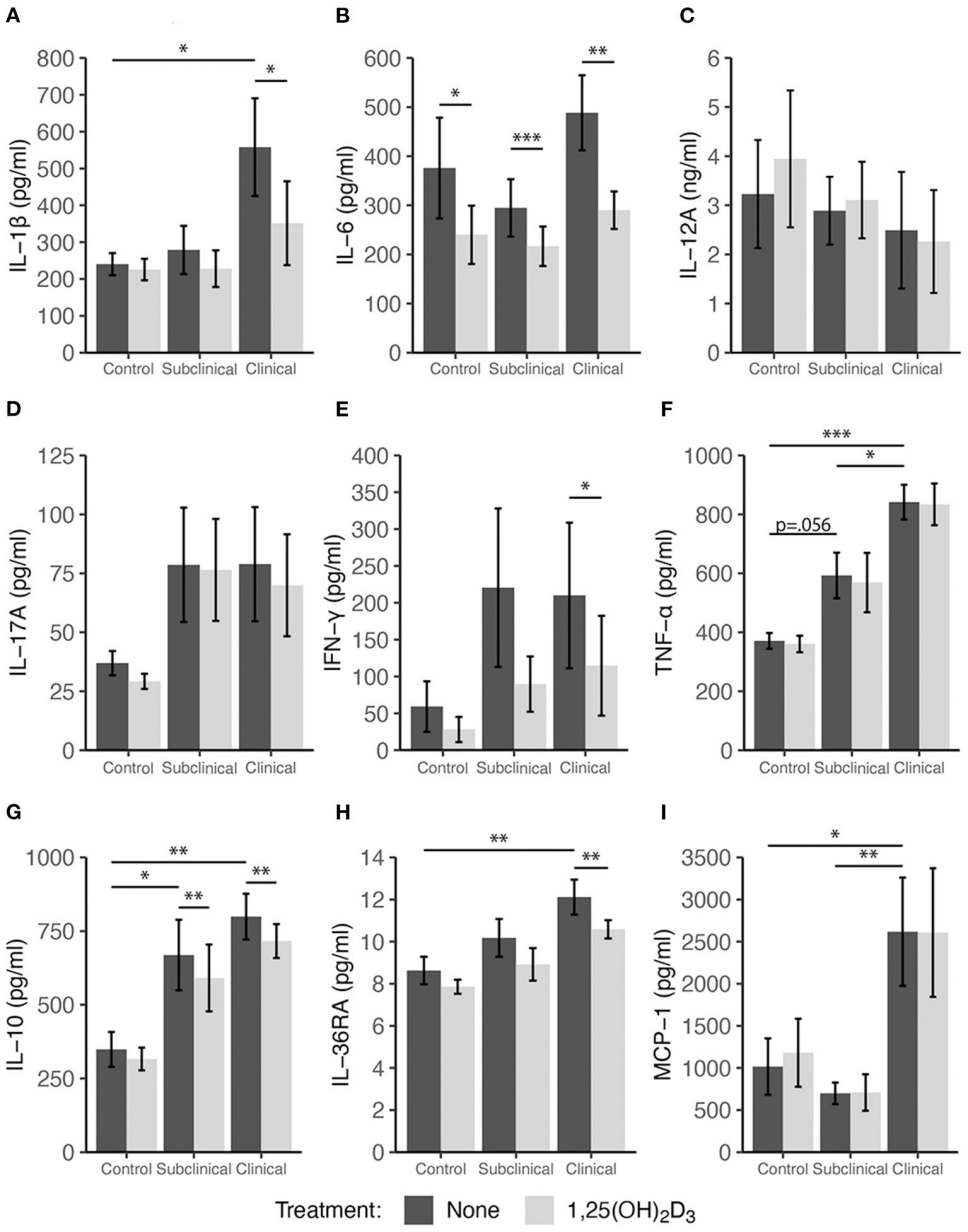
Secretion of cytokines by PBMCs isolated from naturally infected dairy cattle (subclinical *n* = 8, clinical *n* = 8) or non-infected controls (*n* = 8). Peripheral blood mononuclear cells were cultured 24 h with whole cell MAP sonicate (MPS) ± 4 ng/ml 1,25(OH)_2_D_3_. Cell culture supernatants were incubated overnight with cytokine specific magnetic beads for **(A)** IL-1β, **(B)** IL-6, **(D)** IL-17A, **(E)** IFN-γ, **(F)** TNF-α, **(G)** IL-10, **(H)** IL-36RA, and **(I)** MCP-1 supplied in the Milliplex bovine multiplex assay. Fluorescence was measured using the Luminex MAGPIX xMAP instrument and cytokine concentrations were determined by standard curve. Standard ELISA was used to measure **(C)** IL-12A concentrations. Data are presented as the mean ± SE and significance levels are as follows: * < 0.05, ** < 0.01, *** < 0.001.

Stimulation with a whole-cell sonicate of MAP (MPS) increased cytokine secretion by PBMCs in infected cows (both subclinical and clinical) for IL-10, IL-17A, IFN-γ, and TNF-α compared to non-infected control cows (Figure 2). Additionally, secretion of IL-1β, IL-36RA, and MCP-1 was higher (*P* < 0.05) for clinical cows compared to controls (Figure 2). Interestingly, treatment of PBMC cultures with 4 ng/μl 1,25(OH)_2_D_3_ for 24 h resulted in significant decreases in pro-inflammatory cytokines IL-1β (Figure 2A; *P* < 0.05), IL-6 (Figure 2B; *P* < 0.01), and IFN-γ (Figure 2E; *P* < 0.05) for clinically infected animals. Subclinical and non-infected control cows also showed a significant decrease in IL-6 production (Figure 2B; *P* < 0.001 and *P* < 0.05, respectively), with trends toward decreased IFN-γ secretion in both groups (*P* = 0.079 and *P* = 0.074, respectively). IL-36RA, an anti-inflammatory regulator of IL-36, also showed similar patterns with a significant decrease (*P* < 0.01) in clinical cows and a trending decrease in secretion for subclinical cows (*P* = 0.07). Surprisingly, 1,25(OH)_2_D_3_ elicited a significant decrease in IL-10 secretion in clinical (*P* < 0.01) and subclinical (*P* < 0.01) groups. No significant differences were observed within any infection group upon treatment with 1,25(OH)_2_D_3_ for IL-17A, MCP-1, or TNF-α.

**Figure 3:**
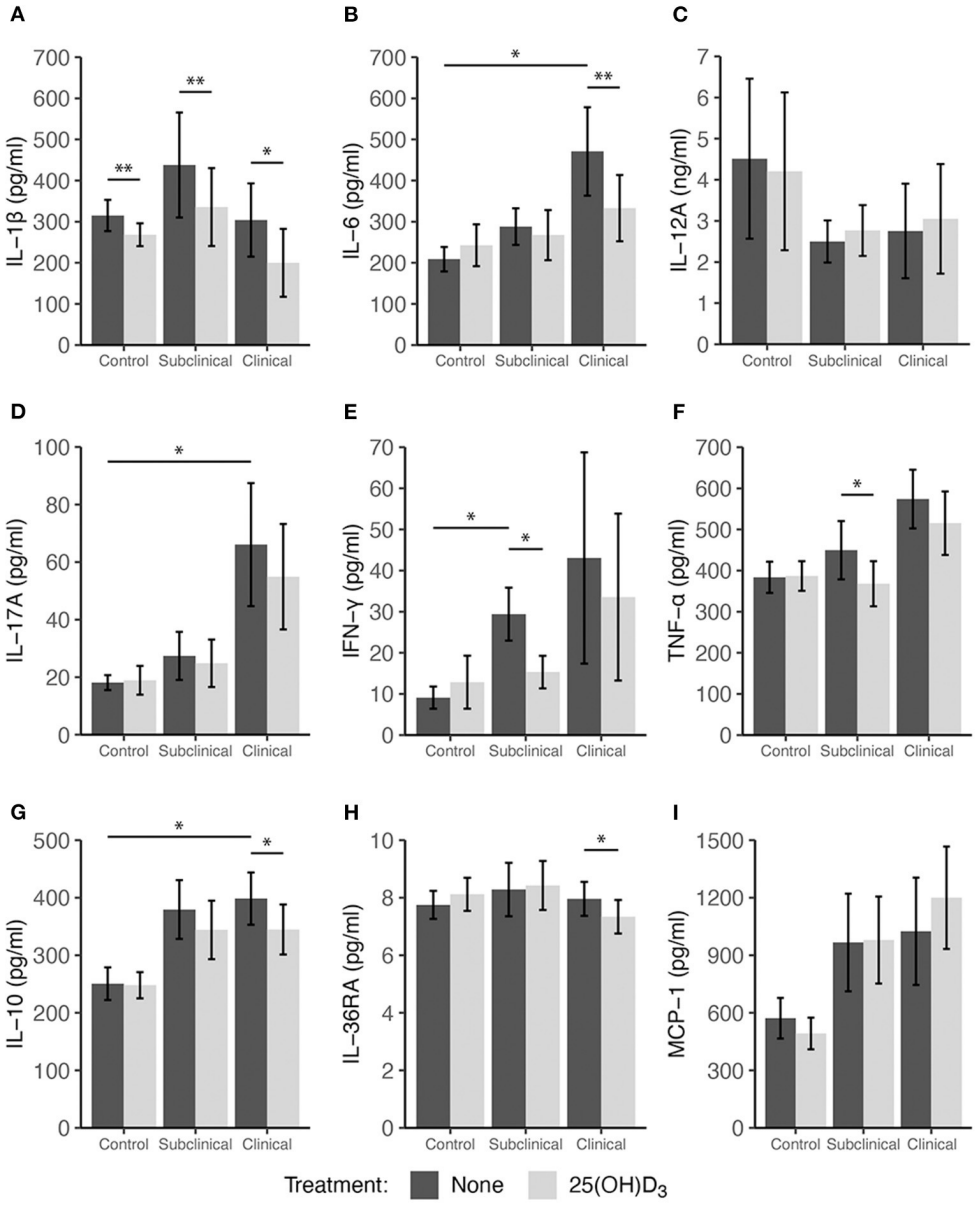
Secretion of cytokines by PBMCs isolated from naturally infected dairy cattle (subclinical *n* = 8, clinical *n* = 8) or non-infected controls (*n* = 8). Peripheral blood mononuclear cells were cultured 24 h with whole cell MAP sonicate (MPS) ± 100 ng/ml 25(OH)D_3_. Cell culture supernatants were incubated overnight with cytokine specific magnetic beads for **(A)** IL-1β, **(B)** IL-6, **(D)** IL-17A, **(E)** IFN-γ, **(F)** TNF-α, **(G)** IL-10, **(H)** IL-36RA, and **(I)** MCP-1 supplied in the Milliplex bovine multiplex assay. Fluorescence was measured using the Luminex MAGPIX xMAP instrument and cytokine concentrations were determined by standard curve. Standard ELISA was used to measure **(C)** IL-12A concentrations. Data are presented as the mean ± SE and significance levels are as follows: * < 0.05, ** < 0.01, *** < 0.001.

Peripheral blood mononuclear cells were also treated with 100 ng/ml 25(OH)D_3_ for 24 h in a sequential study to assess the effects of this vitamin D_3_ precursor on immune cell function. The pattern of cytokine secretion in response to stimulation of cells with MPS alone was reproducible to that observed in the prior experiment, with increased secretion of IL-10, IL-17A, IFN-γ, TNF-α, and MCP-1 observed mainly for infected cows (Figure 3). Comparing non-infected controls to infected cows (subclinical and clinical combined) resulted in a significant increase in MCP-1 production (Figure 3I, *P* = 0.034). The addition of 25(OH)D_3_ to PBMC cultures from clinical animals resulted in a significant decrease in IL-1β (Figure 3A; *P* < 0.05), IL-6 (Figure 3B; *P* < 0.01), and IL-36RA (Figure 3H; *P* < 0.05), an effect that was consistent with that of 1,25(OH)_2_D_3_ treatment as previously described. Control and subclinical animals also exhibited a significant decrease (*P* < 0.01) in IL-1β expression due to 25(OH)D_3_ treatment. Trends for IL-10 secretion were also similar when compared to 1,25(OH)_2_D_3_ treatment but were significantly reduced only in cells isolated from clinical cows (Figure 3; *P* < 0.05). Additionally, decreases in TNF-α secretion were observed in subclinical (*P* < 0.05) and clinical (*P* = 0.065) animals (Figure 3F). In contrast to the effects of 1,25(OH)_2_D_3_, the addition of 25(OH)D_3_ to PBMC cultures resulted in a significant (*P* < 0.05) downregulation of IFN-γ expression only in subclinical animals (Figure 3E). Neither form of vitamin D_3_ had a significant effect on the secretion of IL-12A.

### Cytokine Gene Expression After 1,25(OH)_2_D_3_ or 25(OH)D_3_ Treatment

**Figure 4:**
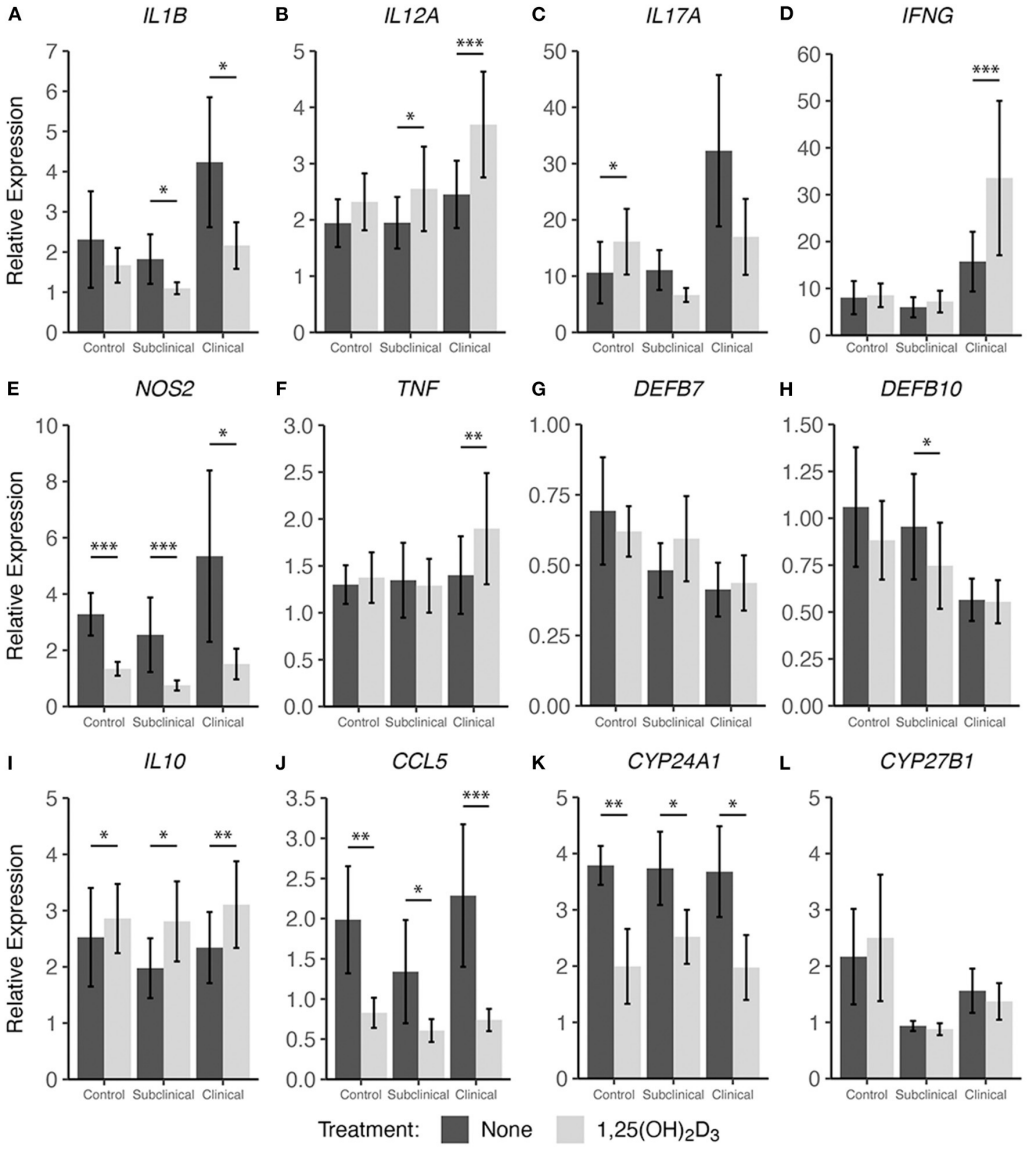
Cytokine gene expression from PBMCs isolated from naturally infected dairy cattle (subclinical *n* = 8 and clinical *n* = 8) or noninfected controls (*n* = 8). Cells were cultured 24 hrs with culture media or whole cell MAP sonicate (MPS) ± 4 ng/ml 1,25(OH)_2_D_3_. Extraction and purification of RNA was performed using Qiagen RNeasy Mini kits and was reverse transcribed with Superscript IV. Gene expression for **(A)** *IL1B*, **(B)** *IL12A*, **(C)** *IL17A*, **(D)** *IFNG*, **(E)** *NOS2*, **(F)** *TNF*, **(G)** *DEFB7*, **(H)** *DEFB10*, **(I)** *IL10*, **(J)** *CCL5*, **(K)** *CYP24A1*, and **(L)** *CYP27B1* were determined using TaqMan assays and were normalized to eukaryotic 18S rRNA reference gene. Data were analyzed using the 2^−Δ*ΔCt*^ method and are presented as the mean relative gene expression (RQ) ± SE compared to each sample's respective non-stimulated (NS) control. Statistics were performed on ΔΔCt values and significance levels are as follows: * < 0.05, ** < 0.01, *** < 0.001.

**Figure 5:**
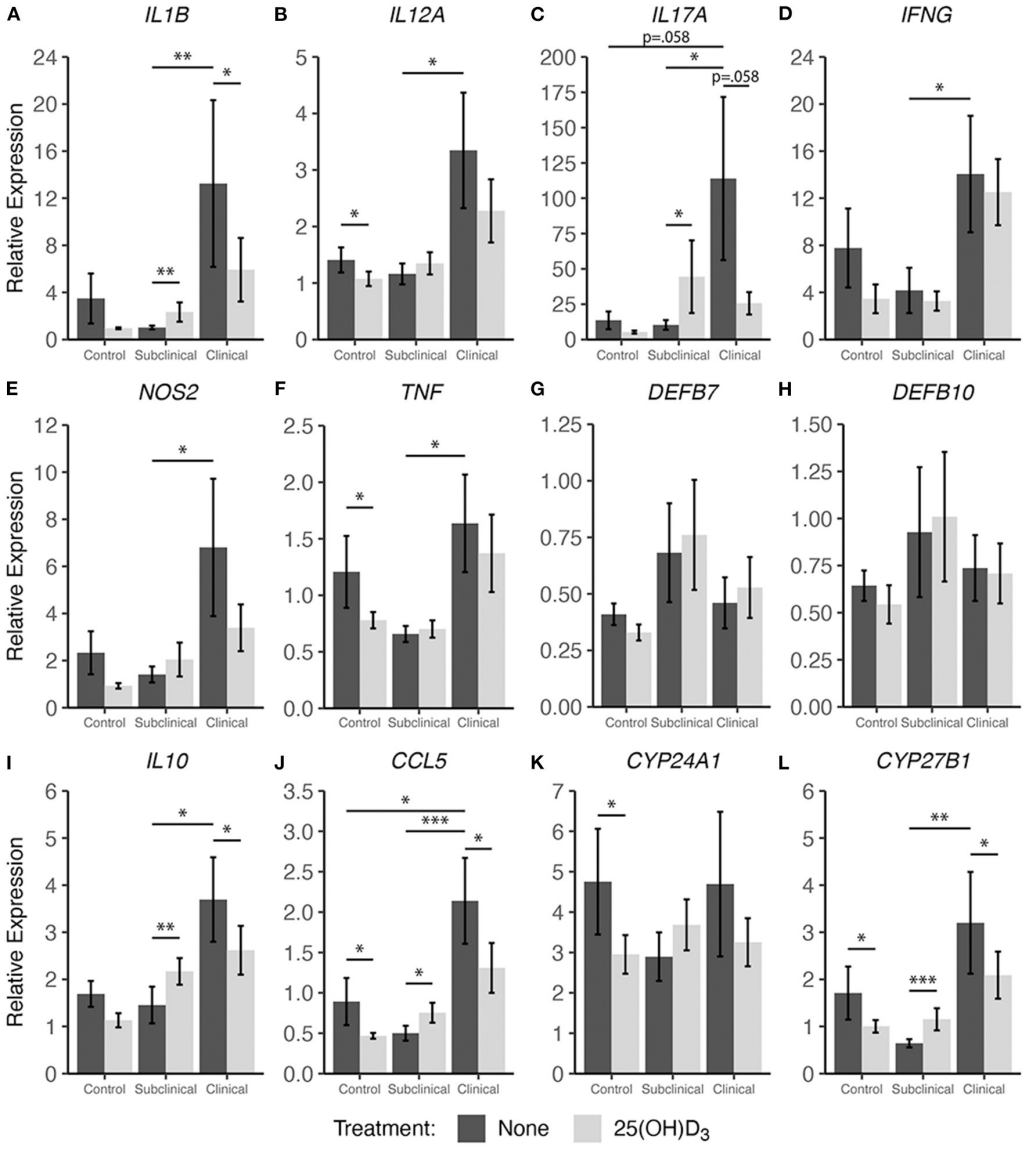
Cytokine gene expression from PBMCs isolated from naturally infected dairy cattle (subclinical *n* = 8 and clinical *n* = 8) or noninfected controls (*n* = 8). Cells were cultured 24 hrs with culture media or whole cell MAP sonicate (MPS) ± 100 ng/ml 25(OH)D_3_. Extraction and purification of RNA was performed using Qiagen RNeasy Mini kits and was reverse transcribed with Superscript IV. Gene expression for **(A)** *IL1B*, **(B)** *IL12A*, **(C)** *IL17A*, **(D)** *IFNG*, **(E)** *NOS2*, **(F)** *TNF*, **(G)** *DEFB7*, **(H)** *DEFB10*, **(I)** *IL10*, **(J)** *CCL5*, **(K)** *CYP24A1*, and **(L)** *CYP27B1* were determined using TaqMan assays and were normalized to eukaryotic 18S rRNA reference gene. Data were analyzed using the 2^−Δ*ΔCt*^ method and are presented as the mean relative gene expression (RQ) ± SE compared to each sample's respective non-stimulated (NS) control. Statistics were performed on ΔΔCt values and significance levels are as follows: * < 0.05, ** < 0.01, *** < 0.001.

In addition to cytokine secretion, we investigated levels of cytokine gene expression from cells accompanying the supernatants from PBMC cultures previously described. For data presentation, relative gene expression was calculated after cells were stimulated with MPS using the respective NS control wells for each cow as the calibrator (Figures 4, 5). Differences in gene expression between groups after treatment with either form of vitamin D_3_ was observed for some pro-inflammatory mediators, with *IL1B* (IL-1β), *IL12A* (IL-12A), *IL17A* (IL-17A), *IFNG* (IFN-γ), and *NOS2* (iNOS) showing patterns of increased transcription in clinical animals compared to subclinicals and control animals. For each of the aforementioned targets, these differences were significant only for subclinical vs. clinical groups within the 25(OH)D_3_ experiment (*P* < 0.05). *TNF* (TNF-α) expression was also significantly upregulated in clinical animals compared to the subclinical group in the 25(OH)D_3_ experiment (Figure 5F; *P* < 0.05). *DEFB7* (β-defensin 7) and *DEFB10* (β-defensin 10) had generally reduced expression for clinical cows compared to the subclinical group, whereas *CCL5* (RANTES), a T cell chemotactic chemokine, was expressed at highest levels in clinical cows and lowest in subclinical cows for both vitamin D_3_ treatment experiments. Significant upregulation of *CCL5* was seen in the 25(OH)D_3_ experiment for untreated PBMCs from clinical cows (Figure 5J) compared to non-infected controls (*P* < 0.05) and subclinical cows (*P* < 0.001). No significant differences were seen between any groups for 24-hydroxylase *CYP24A1* (Figure 5K). The 1α-hydroxylase *CYP27B1* was expressed at higher levels in clinical cows compared to the subclinical group. This difference was significant for the 25(OH)D_3_ experiment (Figure 5L; *P* < 0.01). Subclinical cows also had the lowest levels of transcript expression for this target in both experiments, but the downregulation was not significant compared to non-infected controls.

Interestingly, 1,25(OH)_2_D_3_ treatment significantly upregulated gene expression in clinical animals for multiple pro-inflammatory cytokines including *IL12A* (Figure 4B; *P* < 0.001), *IFNG* (Figure 4D; *P* < 0.001), and *TNF* (Figure 4F; *P* < 0.01). The subclinical group also showed an increase in *IL12A* expression (*P* < 0.05) with 1,25(OH)_2_D_3_ treatment of PBMCs. In contrast, one pro-inflammatory cytokine that demonstrated downregulation due to 1,25(OH)_2_D_3_ addition in the subclinical (*P* < 0.05) and clinical (*P* < 0.05) groups was *IL1B* (Figure 4A). Additional genes that were downregulated after treatment with 1,25(OH)_2_D_3_ were *CCL5* and *NOS2*, the gene for iNOS, both of which showed a significant decrease (*P* < 0.05) across all groups regardless of infection status (Figures 4E,J). The T regulatory cytokine, IL-10, was significantly upregulated in control (*P* < 0.05), subclinical (*P* <0.05), and clinical (*P* < 0.01) animals. Most notably, *CYP24A1* had significantly reduced gene expression in both uninfected (*P* < 0.01) and infected (*P* < 0.05) groups following treatment with 1,25(OH)_2_D_3_. Not surprisingly, 1,25(OH)_2_D_3_ did not affect CYP27B1 gene expression.

Treatment with 25(OH)D_3_ resulted in significant downregulation in clinical animals for the pro-inflammatory mediators *IL1B* (Figure 5A; *P* < 0.05), while *IL17A* and *NOS2* showed a trend of decreased expression (average *P* = 0.097) but no significance (Figures 5C,E). In contrast to 1,25(OH)_2_D_3_ treatment, addition of 25(OH)D_3_ resulted in a significant increase in gene expression for multiple cytokines in subclinical animals including *IL1B* (Figure 5A; *P* < 0.01), *IL17A* (Figure 5C; *P* < 0.05), *IL10* (Figure 5I; *P* < 0.01), *CCL5* (Figure 5J; *P* < 0.05), and *CYP27B1* (Figure 5L; *P* < 0.001). Following 25(OH)D_3_ treatment, control animals showed significant decreases in *IL12A* (Figure 5B; *P* < 0.05), *TNF* (Figure 5F; *P* < 0.05), and *CCL5* (Figure 5J; *P* < 0.05). For this same treatment, a downward trend was observed in clinical animals for *IL1B, IL17A, IFNG, NOS2, DEFB7, DEFB10*, and *IL10* (average *P* = 0.097) although was not significant. *CYP24A1* expression was also significantly reduced in the control group (Figure 5K; *P* < 0.05), while *CYP27B1* showed a significant decrease in transcription activity for both control (Figure 5L; *P* < 0.05) and clinical (*P* < 0.05) groups, and expression was increased in subclinical cows (*P* < 0.001).

### Effects of 25(OH)D_3_ and 1,25(OH)_2_D_3_ on PBMC Marker Expression

**Figure 6:**
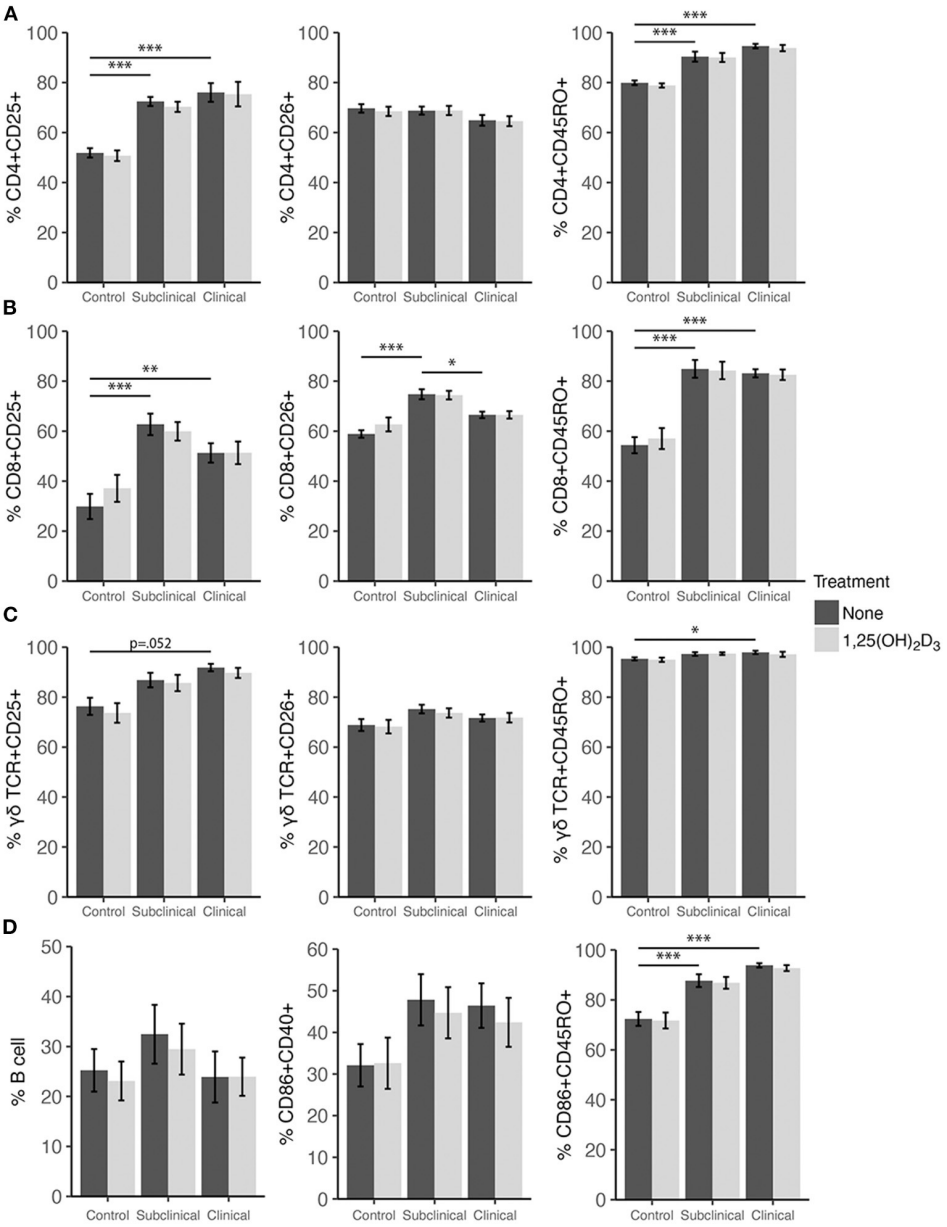
Expression of activation and memory markers on the surface of **(A)** CD4+, **(B)** CD8+, **(C)** γδ T cells, **(D)** monocytes, and total B cells. Peripheral blood mononuclear cells were isolated from naturally infected dairy cattle (subclinical *n* = 8, clinical *n* = 8) or non-infected controls (*n* = 8) and cultured 144 h with whole cell MAP sonicate (MPS) ± 4 ng/ml 1,25(OH)_2_D_3_. Surface markers were detected using fluorescently labeled antibodies and a flow cytometer. Data are presented as the mean percentage ± SE and significance levels are as follows: * < 0.05, ** < 0.01, *** < 0.001.

**Figure 7:**
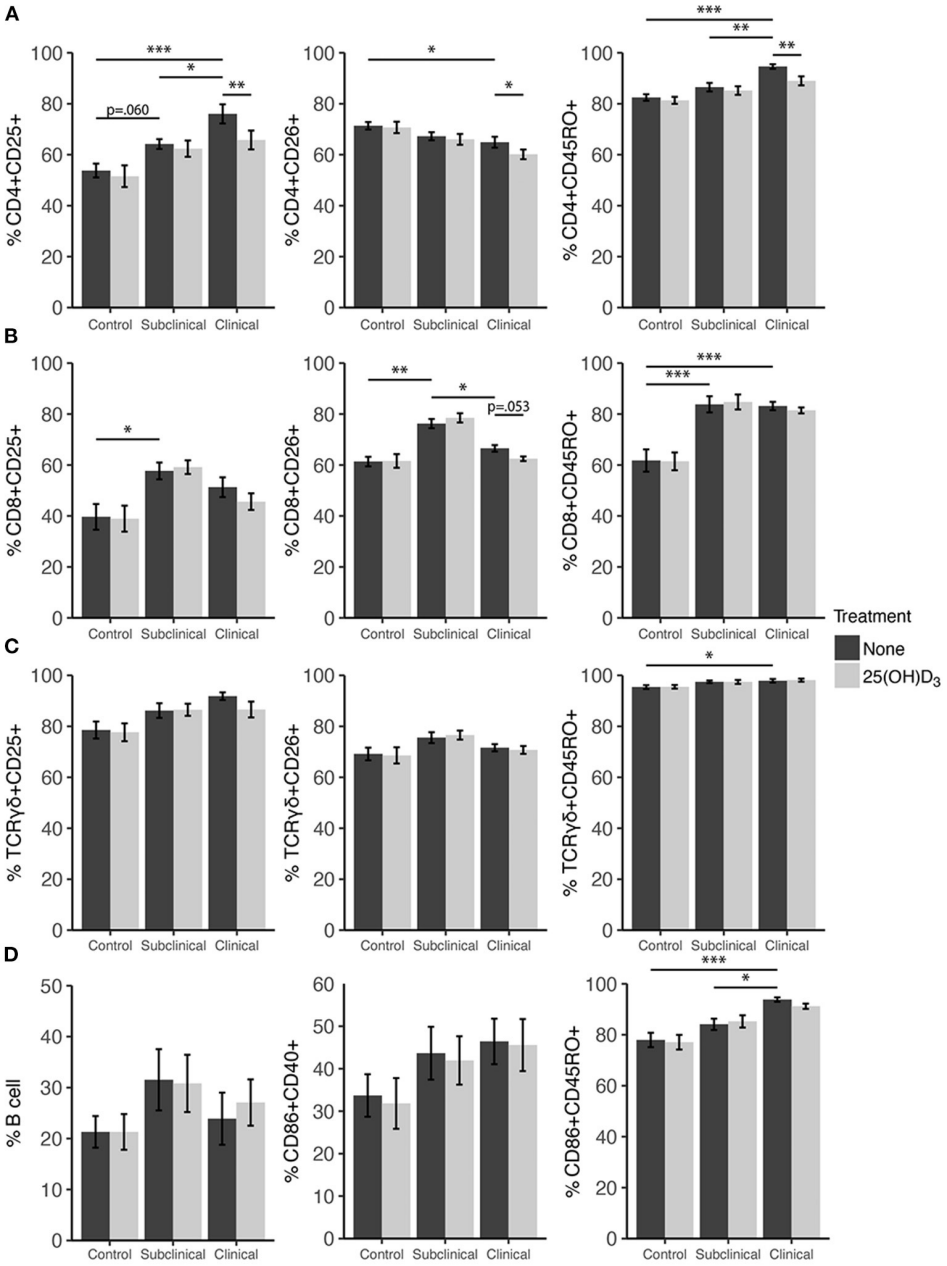
Expression of activation and memory markers on the surface of **(A)** CD4+, **(B)** CD8+, **(C)** γδ T cells, **(D)** monocytes, and total B cells. Peripheral blood mononuclear cells were isolated from naturally infected dairy cattle (subclinical *n* = 8, clinical *n* = 8) or non-infected controls (*n* = 8) and cultured 144 h with whole cell MAP sonicate (MPS) ± 100 ng/ml 25(OH)D_3_. Surface markers were detected using fluorescently labeled antibodies and a flow cytometer. Data are presented as the mean percentage ± SE and significance levels are as follows: * < 0.05, ** < 0.01, *** < 0.001.

Expression profiles for all PBMC subsets were similar for both 1,25(OH)_2_D_3_ and 25(OH)D_3_ treatment experiments (Figures 6, 7). CD4+ cells showed increasing expression levels of activation marker CD25 or memory marker CD45RO with increasing disease severity and was significantly higher in clinical animals compared to controls for both markers (Figures 6A, 7A; *P* < 0.001). In contrast, CD26 expression tended to decrease on CD4+ T cells with increasing disease severity but was found to be significantly reduced in clinical cows compared to uninfected controls in the 25(OH)D_3_ experiment (Figure 7A; *P* < 0.05). CD8+ subpopulations expressing CD25, CD26, or CD45RO were upregulated in infected groups for both vitamin D_3_ experiments, being the highest in subclinical cows. The 25(OH)D_3_ experiment (Figure 7B) showed upregulation was significant between subclinical cows and non-infected controls for CD8+CD25+ (*P* < 0.05), CD8+CD26+ (*P* < 0.01), and CD8+CD45RO+ (*P* < 0.001) subpopulations, and the same comparisons were significant for the 1,25(OH)_2_D_3_ experiment (Figure 6B; *P* < 0.001). Clinical cows in the 1,25(OH)_2_D_3_ experiment had significantly greater CD8+CD25+ cells (*P* < 0.01) compared to non-infected controls, and while CD8+CD26+ expression was also higher it was not significant (*P* = 0.064).

In both vitamin D_3_ experiments (Figures 6, 7), the clinical group had significantly upregulated CD45RO compared to the control group for CD4+ (*P* < 0.001), CD8+ (*P* < 0.001), and γδTCR+ T cell subsets (*P* < 0.05), along with CD86+ subpopulations (*P* < 0.001). Subclinical cows also had significantly greater CD86+CD45RO+ cells compared to controls in the 1,25(OH)_2_D_3_ experiment. Activation markers CD25 and CD26 showed no significant differences in the γδTCR+ subset between groups, however, clinical cows tended to have the highest level of γδTCR+CD25+ cells compared to the control group and this observation was consistent across both vitamin D_3_ experiments (average *P* = 0.099). Activated monocytes, represented in the subset CD86+CD40+ (Figures 6D, 7D), showed no significant differences between infection status groups for either vitamin D_3_ experiment, but tended to be highest in subclinical (average *P* = 0.29) and clinical animals (average *P* = 0.23). There were also no significant differences observed for B cell expression between groups (Figures 6D, 7D).

Effects of vitamin D_3_ treatments on PBMC marker expression were compared within the three infection status groups to ascertain if there was any direct augmentation of immune cell subpopulations. The addition of 1,25(OH)_2_D_3_ to PBMC cultures did not elicit any significant effects for any infection status group on the expression profiles for any of the cell subpopulations investigated (Figure 6). In contrast, differences due to the addition of 25(OH)D_3_ to PBMC cultures were limited but did result in downregulation of activation markers CD25 (*P* < 0.01) and CD26 (*P* < 0.05) on CD4+ T cells for clinical animals (Figure 7A). This same trend was observed in CD8+ expression in clinical cows (Figure 7B) for CD25 and CD26 but did not reach significance (*P* = 0.16 and *P* = 0.053, respectively). Lastly, expression of the memory marker CD45RO was also significantly (*P* < 0.01) decreased on CD4+ T cells following 25(OH)D_3_ treatment (Figure 7A).

## Discussion

As intracellular pathogens, mycobacteria have acquired diverse and complex mechanisms of evading the host immune system, some of which have not been fully characterized. Host recognition of mycobacterial infection by APCs is thought to be initiated by TLR9 binding mycobacterial DNA and TLR2 recognizing cell wall lipoproteins (23–25). Dimerization of TLR2 occurs with TLR1 and TLR6, and increased susceptibility to MAP infection has been reported in cattle containing mutations in TLR1, TLR2, and TLR4. It is plausible that this negatively effects downstream pro-inflammatory cytokine signaling required to control infection. Mycobacterial species, including *M. tb* and MAP, have the ability to utilize intra-endosomal concealment from host immune defenses within macrophages and regulate apoptotic events as a key tool to its success in propagating infection (26, 27). MAP facilitates its survival in the endosome through prevention of the compartment's maturation and acidification, a mechanism which would normally allow for activation of antimicrobial defenses to clear the intracellular pathogen (28).

Infection with MAP revolves around a Th1/Th2 paradigm, with subclinical animals exhibiting characteristics of a Th1 pro-inflammatory response to combat initial infection in which IFN-γ plays an essential role (29). Animals in the advanced clinical stage of disease tend to shift toward a Th2 response, demonstrating increased IL-10 production (30), a progressive loss of cell-mediated cytokine response, and an increase in MAP-specific antibody production that does not actually confer protection (31, 32). Appropriate cross-talk between activated macrophages and T cells is essential to maintain the integrity of host immune defenses; however, MAP can cause both reduced MHCII expression and dysregulation of inflammatory cytokine signaling when compared to less pathogenic species (33, 34).

Vitamin D_3_ is a potent immunomodulatory steroid hormone and its role in pathogenic disease has been of interest, particularly in mycobacterial infections. Previous reports have shown vitamin D_3_ can enhance the antimicrobial capacity of host immune cells (7, 8, 35, 36). The host vitamin D_3_ status may impact the responsiveness of PBMCs to exogenous vitamin D_3_ compounds. Serum 25(OH)D_3_ in cattle has been shown to fluctuate according to season, being higher in the summer months, and may also be impacted by the age of the animal (37). Additionally, serum 25(OH)D_3_ levels necessary for optimal, or even beneficial, immune function have not been concretely established, though the requirement is conservatively estimated to be a minimum of 30 ng/ml, which is slightly greater than the 20 ng/ml threshold required for homeostatic calcium functions (38). Clinical cows in the present study, while not considered deficient, had significantly reduced serum 25(OH)D_3_ levels compared to control and subclinical groups. This could be explained by the general development of severe enteritis as disease progresses, inhibiting nutrient absorption through the gut mucosal surface (10). This group also tended to have more vitamin D_3_ regulatory effects following exogenous treatment of PBMCs. 1,25(OH)_2_D_3_ enhanced gene expression in these clinically infected cows for some pro-inflammatory cytokines (*IL12A, IFNG, TNF*) while 25(OH)D_3_ had no significant effect on these same targets. A lesser studied cytokine in bovine paratuberculosis, IL-36RA, exhibits anti-inflammatory regulatory properties in mice and human studies, and its absence exacerbates inflammatory and pathological skin conditions (39, 40). The significant downregulation of secretion for this cytokine in clinical animals for both forms of vitamin D_3_ is noteworthy and interesting, warranting further study.

Addition of vitamin D_3_ compounds to PBMC cultures did not impact major cell subpopulations, but 25(OH)D_3_ did invoke differences in the expression of activation markers, primarily on CD4+ T cells. These observations were not replicated for 1,25(OH)_2_D_3_ treatment and could be due to the considerably shorter half-life of this form, which has been estimated at 4–6 h in the circulation whereas 25(OH)D_3_ has been estimated to be ~15 days (41). CD25 expression profiles in CD4+ and TCRγδ+ T cells in the present study were consistent with previous reports showing significantly greater numbers in each subset for MAP-infected animals (17). Additionally, an earlier report investigating the effects of intradermal johnin purified protein derivative (PPD) on cows in different stages of infection with MAP showed CD4+CD25+ expression was highest in infected cows (42). CD4+CD25+ regulatory T cells have been shown to be a primary source of IL-10, a key T regulatory cytokine (43), and this subpopulation correlated similarly to *IL10* gene expression and IL-10 cytokine secretion in the current study. Effects of exogenous vitamin D_3_ on PBMC cultures incubated for 6 days with MPS in the current study contrast with a previous observation that 1,25(OH)_2_D_3_ increased CD25 expression on CD4+ and TCRγδ+ T cell subsets in *M. bovis* bacilli Calmette-Guerin (BCG) vaccinated cattle resulting from antigen-specific recall responses to *M. bovis* after 4 days of incubation. Another study investigating proliferation capacity of PBMCs revealed a reduced number of PBMCs expressing CD25 (IL-2 receptor) following incubation with 1 nM of 1,25(OH)_2_D_3_ in a cell culture extending 2–14 days (44). That study demonstrated that it took 6 days in culture to see a significant decrease in CD25 expression.

IL-10 plays a critical role in the ability of MAP to survive within host cells by shifting the nature of the host immune response to Th2, resulting in attenuated pro-inflammatory responses that are critical for the control of infection (45). Blocking TLR2 signaling in bovine monocytes prior to MAP exposure has been shown to reduce IL-10 expression, indicating this may be a pathway utilized by MAP to regulate pro-inflammatory responses (46). Downregulation of TLR2, TLR4, and TLR9 induced by 1,25(OH)_2_D_3_ has been observed in human monocytes, with the reduction of TLR2 and TLR4 corresponding to increased vitamin D receptor (VDR) activity (47, 48). Additionally, increased VDR activity in bovine PBMCs has shown to abrogate expression of IFN-γ following antigen specific responses to *M. bovis* (49). These signaling pathways may be related to the reduction in IL-10, IFN-γ, and IL-6 expression following 1,25(OH)_2_D_3_ or 25(OH)D_3_ treatment observed in the present study. Further detailing the importance of IL-10 function in mycobacterial infection, blocking the mode of action for IL-10 by injecting anti-IL10R monoclonal antibody in a mouse model has been shown to promote clearance of *M. tb* infected lung and spleen tissue (50). Additionally, neutralizing anti-IL-10 antibody enhanced MAP-specific recall response for gene expression of some pro-inflammatory cytokines from PBMC cultures, including IFN-γ, IL-12, and IL-1β (43). MAP infected animals in the present study consistently expressed higher levels of IL-10 secretion as expected, but pro-inflammatory responses were still elevated as well. Curiously, treatment with either form of vitamin D_3_ resulted in a significant reduction in IL-10 secretion for MAP infected groups but had no effect on control animals. Gene expression for the 25(OH)D_3_ treatment experiment was also significantly downregulated in clinical cows but upregulated in subclinicals, while 1,25(OH)_2_D_3_ treatment upregulated *IL10* expression for all groups.

The presentation of *CYP24A1, CY27B1*, and *DEFB7* and *DEFB10* data herein are novel for cattle naturally infected with MAP, allowing us to correlate the expression of these genes with responses to vitamin D_3_ supplementation (*CYP* and *DEFB*) and recall responses to antigen stimulation (*DEFB*). Expression of *DEFB7* and *DEFB10* in the present study was not affected by natural infection of cattle with MAP. This is surprising because one might expect an increased expression of defensins in recall responses to antigen in infected cows. However, a trend toward decreased *DEFB* expression was observed in clinical cows, which would correlate with a reduced ability to control infection. Previous work has shown intestinal β-defensin expression tapers by 12 h post infection of healthy calf ileum (51). Additionally, somatic cell expression of defensins in a mastitis model showed 25(OH)D_3_ treatment having no effect on *DEFB7* and *DEFB10* expression (52) while 1,25(OH)_2_D_3_ treatment increased *DEFB7* expression (53).

In the present study, levels of *CYP24A1* and *CYP27B1* were not affected by infection status, except for lower *CYP27B1* expression for subclinical cows compared to clinicals. Addition of 1,25(OH)_2_D_3_ to non-stimulated bovine monocyte and PBMC cultures has been shown to induce *CYP24A1* expression (4, 5), and following antigen activation of the cells 1,25(OH)_2_D_3_ induced upregulation is dampened. These trends in expression were also observed in the present study (data not shown). Our study also saw MPS activation increase *CYP24A1* expression relative to non-stimulated controls and addition of 1,25(OH)_2_D_3_ dampen its upregulation for all infection status groups. In contrast, 25(OH)D_3_ has not been shown to induce significant changes in expression in bovine PBMCs compared to non-stimulated control cells, but stimulation with antigen and 25(OH)D_3_ treatment resulted in a slight increase in expression compared to antigen stimulation alone (5). Our study presents a slight reduction in *CYP24A1* expression following addition of 25(OH)D_3_ to MPS stimulated cells in control cows, with no significant changes seen in other groups. These results show that both forms of vitamin D_3_ may regulate *CYP24A1* transcripts in healthy control cows, but the active metabolite is needed in excess to modulate expression in infected cows. An increase in *CYP27B1* expression upon stimulation with LPS antigen and vitamin D_3_ treatment in bovine monocytes from healthy dairy cattle has been reported (4), which is corroborated by LPS-induced increase in *CYP27B1* observed in the current study for healthy control cows (Supplementary Figure 1).

Work in cattle has shown contrasting patterns of expression for *NOS2* and *CCL5*, perhaps highlighting the importance of context within cellular signaling and antigen specific responses. Bovine monocytes demonstrate increased *NOS2* and *CCL5*/RANTES expression following LPS activation and treatment with 1,25(OH)_2_D_3_ or 25(OH)D_3_ (4), which contrasts with the observations in our study showing PBMCs stimulated with LPS (Supplementary Figure 1) or MPS and treated with 1,25(OH)_2_D_3_ resulted in a general decrease in *NOS2* and *CCL5* expression. These two genes had very similar expression patterns when treated with 25(OH)D_3_, decreasing in clinical and control groups but being upregulated in subclinical cows. Studies using human and mice models have reported a 1,25(OH)_2_D_3_ induced upregulation of *NOS2*; however this discrepancy may be due in part to species differences. For example, antimicrobial activity of human monocytes against *M. tuberculosis* elicits upregulation of the single cathelicidin gene found in humans (LL-37) (36). Current data show cattle have 11 cathelicidin genes, and the few with potential vitamin D response elements have been found not to be influenced by vitamin D treatment (4, 38). It is therefore plausible other mechanisms of immune regulation may be different between species and require further investigation. Moreover, additional work in our lab has shown 1,25(OH)_2_D_3_ treatment of monocyte derived macrophage (MDM) and PBMC co-cultures results in significant downregulation of *NOS2*, but no effects were observed for this treatment on *CCL5* (54). Peripheral blood mononuclear cells from calves vaccinated for *M. bovis-*BCG showed increased *CCL5* expression following activation with *M. bovis* PPD and treatment with 1,25(OH)_2_D_3_ or 25(OH)D_3_ (5). In cattle affected by subclinical mastitis, intra-mammary treatment with 1,25(OH)_2_D_3_ has shown no effect on *CCL5* expression by total milk somatic cells over the course of 72 h (53). Furthermore, a LPS induced mastitis model has shown intra-mammary treatment with 25(OH)D_3_ results in a reduction in *CCL5* expression in macrophages but has no effect on expression in neutrophils (52).

Taken together, the addition of exogenous 25(OH)D_3_ or 1,25(OH)_2_D_3_ appeared to have similar biological effects on PBMC cytokine secretion and gene expression overall, commonly decreasing both pro-inflammatory and regulatory cytokine secretion. An exception for 1,25(OH)_2_D_3_ treatment, however, was seen in the upregulation of *IL10* expression in all groups of cows and the significant increase in pro-inflammatory transcripts of *IL12A, IFNG*, and *TNF* in clinically infected cows. This may indicate that addition of this active vitamin D_3_ analog in concentrations exceeding normal physiological levels exerts compensatory mechanisms for some pro-inflammatory cytokines in animals with low circulating 25(OH)D_3_ resulting from a severe disease state but may overall stimulate regulatory effects through increasing *IL10* transcripts. Significant downregulation was seen with 25(OH)D_3_ treatment for some cell surface markers in CD4+ subpopulations; however, as stated previously this may be due to the longer biological half-life it possesses compared to 1,25(OH)_2_D_3_, which may have shown transient effects prior to day 6 when data was acquired for this study_._

In conclusion, these data provide evidence that vitamin D_3_ plays a role in cytokine regulation for MAP-specific antigen recall responses within PBMCs from MAP infected dairy cattle. Host responses can vary depending on the cow's infection status and appears to coincide with serum 25(OH)D_3_ levels. This study demonstrates the complexity of the Th1/Th2 immune response switch characteristic to Johne's disease. The present study also provides a foundation for further characterization of vitamin D_3_ effects upon regulatory mechanisms within the bovine immune system. Investigation into vitamin D_3_ effects on TLR and VDR expression, along with their downstream effects on MAP viability would be a valuable contribution to the field. Future work incorporating a time series or incubations extending past the 24 h used in this study may provide more insights on the differences between both cytokine secretion and mRNA transcript levels, and also the length of time necessary for each vitamin D_3_ analog to fully demonstrate any effects on immune system signaling.

## Data Availability Statement

The original contributions presented in the study are included in the article/Supplementary Material, further inquiries can be directed to the corresponding author/s.

## Ethics Statement

The animal study was reviewed and approved by National Animal Disease Center Animal Care and Use Committee.

## Author Contributions

JS and TW conceived experimental design. TW performed experiments, data analysis, and first draft manuscript preparation. JS, TW, and SM contributed to manuscript revisions. All authors contributed to the article and approved the submitted version.

## Funding

This study was funded through USDA-ARS, CRIS Project 5030-32000-221.

## Conflict of Interest

The authors declare that the research was conducted in the absence of any commercial or financial relationships that could be construed as a potential conflict of interest.

## Publisher's Note

All claims expressed in this article are solely those of the authors and do not necessarily represent those of their affiliated organizations, or those of the publisher, the editors and the reviewers. Any product that may be evaluated in this article, or claim that may be made by its manufacturer, is not guaranteed or endorsed by the publisher.
