# Supplementary material for: Effects of 1,25-Dihydroxyvitamin D3 and 25-Hydroxyvitamin D3 on PBMCs From Dairy Cattle Naturally Infected With Mycobacterium avium subsp. paratuberculosis
Source: Front Vet Sci. 2022 Feb 8;9:830144. doi: 10.3389/fvets.2022.830144 (PMC8861496; doi:10.3389/fvets.2022.830144)
Supplement: Supplementary file 1 [file Image_1.pdf]

Supplementary Material

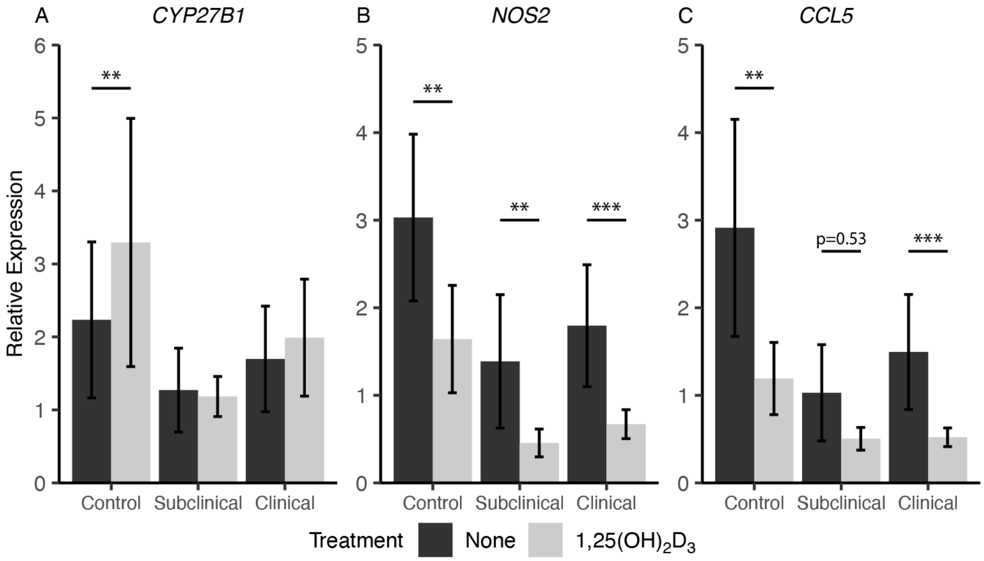

Supplementary Figure 1. (A) *CYP27B1*, (B) *NOS2*, and (C) *CCL5* gene expression from PBMCs isolated from naturally infected dairy cattle (subclinical n=8, clinical n=8) or noninfected controls (n=8). Cells were cultured 24 hrs with culture media or 1.0 µg/ml LPS ± 4 ng/ml 1,25$(OH)_2D_3$. Extraction and purification of RNA was performed using Qiagen RNeasy Mini kits and was reverse transcribed with Superscript IV. Gene expression was determined using TaqMan assays and was normalized to eukaryotic 18S rRNA reference gene. Data were analyzed using the $2^{-\Delta\Delta Ct}$ method and are presented as the mean relative gene expression (RQ) ± SE compared to each sample's respective non-stimulated (NS) control. Statistics were performed on ΔΔCt values and significance levels are as follows: * <.05, ** <.01, *** <.001.
